# CSF Extracellular Vesicle Aβ42 and Tau/Aβ42 Ratio Are Associated with Cognitive Impairment in Older People with HIV

**DOI:** 10.3390/v16010072

**Published:** 2023-12-31

**Authors:** Debjani Guha, Vikas Misra, Sukrutha Chettimada, Jun Yin, Dana Gabuzda

**Affiliations:** 1 Department of Cancer Immunology and Virology, Dana-Farber Cancer Institute, Boston, MA 02215, USA; 2 Department of Neurology, Harvard Medical School, Boston, MA 02115, USA

**Keywords:** HIV, cognitive impairment, cerebrospinal fluid, amyloid, Tau, extracellular vesicles

## Abstract

HIV-associated neurocognitive disorders (HAND) remain prevalent despite viral suppression on antiretroviral therapy (ART). Older people with HIV (PWH) are also at risk for amnestic mild cognitive impairment (aMCI) and Alzheimer’s disease (AD). β-amyloid (Aβ) and Tau biomarkers are associated with aMCI/AD, but their relationship to HAND is unclear. Given the role of extracellular vesicles (EVs) in age-related neurological disorders, we investigated soluble and EV-associated Aβ42, total Tau, NFL, GFAP, ICAM-1, VCAM-1, and CRP in relation to cognitive impairment in PWH. Plasma and CSF EVs were isolated from 184 participants (98 PWH on ART and 86 HIV− controls). Biomarkers were measured using Meso Scale Discovery assays. The median age of PWH was 53 years, and 52% were diagnosed with mild forms of HAND. PWH had increased plasma NFL (*p* = 0.04) and CSF Aβ42 (*p* = 0.0003) compared with HIV− controls but no significant difference in Tau or EV-associated forms of these markers. CSF EV Aβ42 was decreased (*p* = 0.0002) and CSF EV Tau/Aβ42 ratio was increased (*p* = 0.001) in PWH with HAND vs. no HAND, while soluble forms of these markers showed no significant differences. Decreased CSF EV Aβ42 (*p* < 0.0001) and an increased CSF EV Tau/Aβ42 ratio (*p* = 0.0003) were associated with lower neurocognitive T scores in age-adjusted models; an optimal model included both CSF EV Aβ42 and plasma NFL. Levels of soluble, but not EV-associated, ICAM-1, VCAM-1, and CRP were increased in PWH with HAND vs. no HAND (*p* < 0.05). These findings suggest that decreased Aβ42 and an increased Tau/Aβ42 ratio in CSF EVs are associated with cognitive impairment in older PWH, and these EV-associated biomarkers may help to distinguish aMCI/AD from HIV-related cognitive disorders in future studies.

## 1. Introduction

Combination antiretroviral therapy (ART) has increased the longevity of people with HIV (PWH), but the impact of aging on cognitive health remains a major concern [1,2]. HIV-associated neurocognitive disorders (HAND), consisting of asymptomatic neurocognitive impairment (ANI), mild neurocognitive disorder (MND), and HIV-associated dementia (HAD), occur in 20% to 50% of PWH, with prevalence varying by age, viral suppression, comorbidities, and lifestyle factors [1,3]. Although HAD is uncommon in PWH on current ART, milder forms of HAND remain prevalent [3,4]. The mechanisms involved in HAND are heterogeneous, including ongoing viral replication in the CNS, neuroinflammation, oxidative stress, vascular disease, blood–brain barrier (BBB) dysfunction, ART-related neurotoxicity, and substance use [3,4,5]. Due to persistent inflammation, cerebrovascular disease, and small-vessel disease-related brain injury, PWH are also at increased risk of premature brain aging [1,6,7,8,9,10]. However, the relative risk of amnestic mild cognitive impairment (aMCI) and Alzheimer’s disease (AD) in PWH remains unclear [11,12].

Neurofilament light chain (NFL) is an established marker of neuronal injury associated with cognitive impairment in PWH [13,14,15,16]. However, it is a nonspecific marker that reflects axonal injury in many neurological disorders. β amyloid (1–42) (Aβ42) aggregates and hyperphosphorylated Tau-containing neurofibrillary tangles are hallmarks of AD that are detected in the brains of some older PWH [17,18,19,20]. An increased CSF Tau/Aβ42 ratio is a biomarker associated with risk of aMCI and AD; increased CSF Tau reflects its release from degenerating neurons while decreased CSF Aβ42 reflects higher amyloid deposition in the brain [21,22,23,24]. Most studies of aMCI/AD-related biomarkers according to HIV serostatus have not detected a significant increase among PWH compared to the general population [11,20,25,26,27]. Moreover, early neuroinflammation in PWH might have protective effects against Aβ and Tau deposition in the brain [28]. Nonetheless, altered Aβ metabolism, BBB dysfunction, vascular disease, and ART neurotoxicity have the potential to accelerate development of aMCI/AD-related pathology in PWH [1,6,18,29,30]. Furthermore, the duration of HIV infection may be a risk factor for increased Aβ deposition in the brain [31]. These findings indicate the need for biomarkers that can help to distinguish aMCI/AD from other biologically defined subtypes (biotypes) of cognitive impairment in PWH [11,12,25,32].

Extracellular vesicles (EVs) are a new source of biomarkers in various neurological disorders, including HAND and AD [16,33,34,35]. EVs are classified as exosomes (50–150 nm) or microvesicles (200 nm–1 μm) and carry proteins, lipids, and nucleic acids from parental cells to recipient cells to mediate physiological and pathological functions. EV-associated biomarkers can provide different information than soluble biomarkers because EVs originate from cellular compartments (i.e., endosomal multivesicular bodies or plasma membranes) and may therefore reflect cellular processes/pathobiology in ways not reflected by soluble biomarkers. Previous studies have detected aggregated forms of Aβ and p-Tau in exosomes/EVs [33,36,37,38]. Furthermore, EVs may play a functional role in the spreading of Tau pathology [39,40]. Although some studies have detected an association between elevated Aβ42 in plasma neuronal-derived EVs and aMCI/AD [35,41,42], the prognostic significance of EV-associated biomarkers in aMCI/AD-related disorders remains unclear.

EVs play functional roles in HIV pathogenesis and carry proteins with the potential to impact cognitive impairment in PWH [16,43,44,45]. CSF EVs are increased in HAND and carry cellular proteins related to immune activation/inflammation, stress responses, glial activation, and BBB [16,34,46]. Accordingly, EV-associated biomarkers may help to distinguish HAND from aMCI/AD and other neurological disorders by providing information that reflects disease-specific cellular processes/pathobiology. Here, we investigated the association of EV biomarkers related to CNS injury (Tau, Aβ42, GFAP) and vascular injury (ICAM-1, VCAM-1, CRP) with HAND in PWH on suppressive ART.

## 2. Materials and Methods

### 2.1. Study Participants

Plasma and CSF samples from 184 participants (*n* = 98 PWH on ART and *n* = 86 HIV− controls) were collected between 2006 to 2016, including 66 paired plasma and CSF samples from PWH; the remaining 118 participants had only a plasma (*n* = 73) or CSF (*n* = 45) sample. HIV+ samples were from the National NeuroAIDS Tissue Consortium (NNTC) and CNS HIV Anti-Retroviral Therapy Effects Research (CHARTER) study [47,48]. All participants were enrolled with written informed consent and institutional review board approval at each site. Eligible PWH participants were aged 30 years and older, had been taking 3 or more ART drugs for at least 1 year, and were virally suppressed (plasma viral load [VL] < 200 copies/mL). PWH with HAD or neuropsychological impairment due to other causes (NPI-O) were excluded because HAD diagnoses are rare in the current ART era and NPI-O reflects confounding diagnoses. Plasma and CSF samples from HIV− individuals without a diagnosed neurological disease (from Bioreclamation LLC, Westbury, New York, NY, USA) were group-matched for age, gender, and race.

### 2.2. Assessment of Cognitive Function and HAND Diagnoses

PWH were administered a comprehensive neuropsychological test battery designed to assess 7 cognitive domains (abstraction/executive function, speed of information processing, attention/working memory, learning, memory, verbal fluency, and motor function). Raw test scores were transformed into age-, gender-, race-, and education-adjusted T scores for each domain, and global T scores were generated from the domain T scores as described [47,49]. T scores correlate negatively with the severity of neurocognitive impairment, with values below 40 (corresponding to 1 standard deviation of 10 below a normalized mean of 50) signifying impairment. Deficits in speed of information processing, attention/working memory, memory, and motor domains are common in HAND, while specific deficits in learning/memory are more characteristic of aMCI [11,12,50]. HAND diagnoses of ANI and MND were determined using established criteria [51] based on neurocognitive testing and neurological evaluation. PWH were classified as cognitively impaired if they had a HAND diagnosis of ANI or MND, corresponding to mild cognitive impairment without or with interference in everyday function, respectively. Medical comorbidities were classified based on self-reporting, medical records, and/or a review of medications and lab values as described [52].

### 2.3. Isolation and Characterization of Plasma and CSF Extracellular Vesicles

To isolate plasma EVs, plasma samples (300 µL) were centrifuged at 1500× *g* and 10,000× *g* for 10 and 20 min, respectively, to remove cell debris. Supernatants were applied onto Izon qEV 35 nm size-exclusion columns (Izon Science, Medford, MA USA), and 0.5 mL fractions were collected using an Izon qEV automated fraction collector. Two fractions containing the majority of EVs, based on transmission electron microscopy (TEM) and nanoparticle tracking analysis (NTA), were pooled and concentrated using Amicon Ultra-4 10 kDa molecular weight centrifugal filter units to a final volume of 50 µL. To isolate CSF EVs, CSF samples (300 µL) were centrifuged at 3000× *g* to remove cell debris, and the supernatants were incubated overnight at 4 °C with ExoQuick (System Biosciences, Palo Alto, CA, USA) according to the manufacturer’s protocol. The suspensions were centrifuged at 1500× *g* for 30 min, and the CSF EV pellets were suspended in 30 µL PBS. Plasma and CSF EVs were diluted 1:10,000 and 1:5000, respectively, for TEM and NTA, or lysed with RIPA buffer (Triton X-100 1%, NaCl 150 mmol/L, sodium deoxycholate 0.5%, Tris-HCL 50 mmol/L, SDS 0.1%, pH 7.4) for biomarker assays or immunoblotting. EV concentrations and sizes were measured by NTA (Particle Metrix ZetaView, Mebane, NC, USA). TEM was performed using a TecnaiG2 Spirit BioTWIN instrument (FEI company, Hillsboro, OR, USA) equipped with an AMT 2 k CCD camera (Harvard University TEM core, Boston, MA, USA).

### 2.4. Meso Scale Discovery Assays

Plasma and CSF samples were centrifuged at 1200 rpm for 5 min at 4 °C to remove cells and debris, and the supernatants were aliquoted and stored at −80 °C. CNS injury (NFL, total Tau, Aβ42), glial activation (GFAP), and vascular injury (ICAM-1, VCAM-1, CRP) markers were measured in the plasma and CSF samples or lysed plasma and CSF EVs using the Meso Scale Discovery (MSD) platform (Rockville, Maryland). CNS injury and glial activation markers were measured using R-Plex custom panels. Vascular injury markers were measured using the V-Plex Vascular Injury Panel 2 kit. Plates were read using the MESO SECTOR S 600 imager and the data analyzed using MSD Discovery Workbench 4.0 software. When biomarker levels were not detectable, participants were assigned the lowest detected value. EV biomarker concentrations were normalized for protein concentration.

### 2.5. Proteinase K Treatment and Immunoblotting of CSF EVs

Isolated CSF EVs were incubated with or without 0.5 mg/mL proteinase K (PK) (Life Technologies, Carlsbad, CA USA) at 37 °C for 10 min before neutralization of PK activity by adding 5 mM PMSF. Frontal cortex gray matter tissue samples (~30 mg) obtained at autopsy from 2 HIV+ individuals over age 60 with HAND were homogenized and lysed in RIPA buffer with protease inhibitors. Proteins in the CSF EV and brain tissue lysates were separated on SDS-polyacrylamide gels and transferred onto PVDF membranes. Blots were probed with primary antibodies against Aβ42 (Cell Signaling Technology, Danvers, MA, USA), Tau (Tau 5, BioLegend, San Diego, CA, USA), CD9 (Santa Cruz Biotechnology, Dallas, TX, USA), CD63 (EXOAB-KIT-1; System Biosciences), HSP70 (Cell Signaling Technology), and Flotillin-1 (BD Biosciences, Franklin Lakes, NJ, USA) overnight at 4 °C. Secondary antibodies were incubated for 1.5 h at room temperature. The signals were developed using enhanced chemiluminescence and captured using the Biorad ChemiDoc Imaging System.

### 2.6. Data Analysis

Demographics, clinical covariates, and biomarker levels were compared between groups using chi-square or Fisher’s exact test for categorical variables and the Mann–Whitney U test for continuous variables. Relationships between continuous variables were analyzed by Spearman’s rank correlation. Associations between log2-transformed biomarkers and global T scores were analyzed in linear regression models adjusted for age. A two-sided *p*-value less than 0.05 was considered statistically significant. Of primary interest was the exploration of possible associations between EV-associated Tau and Aβ42 and HAND, and to examine whether EV-associated Tau and Aβ42 have stronger associations with HAND than soluble forms of these and other markers examined. Thus, *p*-values were not adjusted for multiple comparisons. Backward elimination, starting with the full model (four biomarkers, age, gender, and race), followed by the sequential elimination of variables with the least significant contribution was used to develop an optimal regression model based on the lowest Akaike information criterion (AIC) value. Analyses were performed using GraphPad Prism version 9 and the R Stats Package, version 4.2.1.

## 3. Results

### 3.1. Study Participants

Demographic and clinical characteristics of the study population are shown in Table 1. The study cohort included 98 PWH on ART (median age, 53 years [IQR 47–59]; 84% male; 69% white; median duration of HIV infection, 15 years [5,6,7,8,9,10,11,12,13,14]) and 86 HIV− controls, matched for demographics. Plasma and CSF VL were suppressed (<200 and <50 copies/mL, respectively) in 96% of PWH, and their median CD4 and nadir CD4 counts were 540 and 84 cells/µL, respectively. Fifty-two percent were diagnosed with mild forms of HAND (53% ANI, 47% MND). Cerebrovascular and/or cardiovascular diseases (CeVD/CVD) (e.g., coronary artery disease, myocardial infarction, stroke, brain infarcts, lacunes; hereafter termed vascular disease) were more prevalent in PWH with HAND compared with those with no HAND (*p* = 0.005), while age, HIV-related parameters, diabetes, hypertension, and hyperlipidemia showed no significant differences.

### 3.2. Characterization of Plasma and CSF Extracellular Vesicles

**Figure 1:**
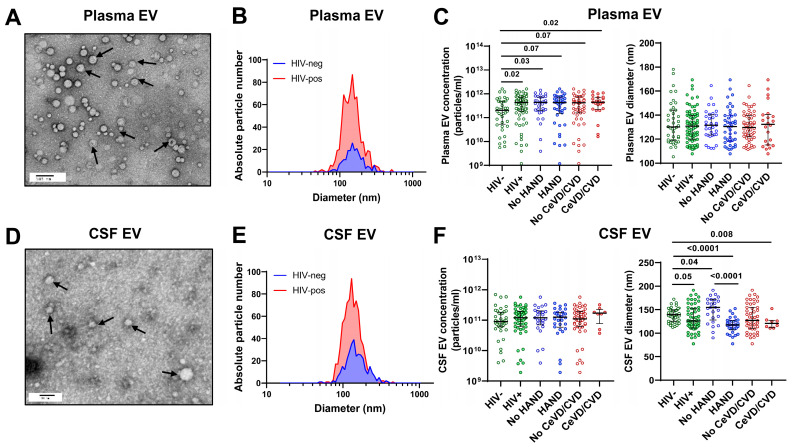
Characterization of extracellular vesicles isolated from plasma and CSF. (**A**) Transmission electron microscopy (TEM) images of plasma EVs isolated from a representative HIV− individual. Arrows indicate EVs. Scale bars = 100 nm. (**B**) Size distribution histograms of plasma EVs by nanoparticle tracking analysis (NTA) of representative HIV− and HIV+ individuals. (**C**) Association of plasma EV concentration and size with HIV status, HAND, and vascular disease. (**D**) TEM images of CSF EVs isolated from a representative HIV− individual. (**E**) Size distribution histograms of CSF EVs by NTA of representative HIV− and HIV+ individuals. (**F**) Association of CSF EV concentration and size with HIV status, HAND, and vascular disease. Exosome markers CD63, CD9, and Flotillin-1 were detected by immunoblotting of plasma and CSF EVs (Supplemental Digital Content S1). Medians and IQRs are indicated as horizontal and vertical lines, respectively. Statistical significance was calculated using the Mann–Whitney U test; significant differences (*p* < 0.05) are indicated.

Plasma and CSF EV fractions were isolated and characterized by TEM, NTA, and immunoblotting for exosome markers (Figure 1). TEM revealed vesicles corresponding to the size of exosomes (Figure 1A,D). NTA showed that most particles were 30–150 nm in diameter, with a peak at 100–120 nm (Figure 1B,E). Immunoblotting detected the exosome markers CD9, CD63, and Flotillin-1 in both plasma and CSF EV fractions (Supplemental Digital Content S1), indicating an enrichment of exosomes. Plasma and CSF EV concentration and size were compared between groups by HIV status, HAND, and vascular disease (CeVD/CVD) (Figure 1C,F). Most plasma and CSF EVs were in the 90–150 nm size range of exosomes. Plasma EV concentrations were higher in PWH vs. HIV− controls (*p* = 0.02) but similar between groups by HAND or vascular disease status (Figure 1C). CSF EV concentrations were similar across groups irrespective of HIV status, HAND, or vascular disease, with median concentrations around 10^11^ particles/mL (Figure 1F). While plasma EVs showed no significant difference in size between groups, CSF EVs showed a decreased median size in PWH with HAND vs. no HAND (*p* < 0.0001) and PWH vs. HIV− controls (*p* = 0.05) (Figure 1C,F), possibly reflecting an increased production of exosomes (i.e., smaller-diameter EVs) by activated microglia/astrocytes and/or injured neurons.

### 3.3. Association of CSF EV Aβ42 Levels and Tau/Aβ42 Ratio with HAND

**Figure 2:**
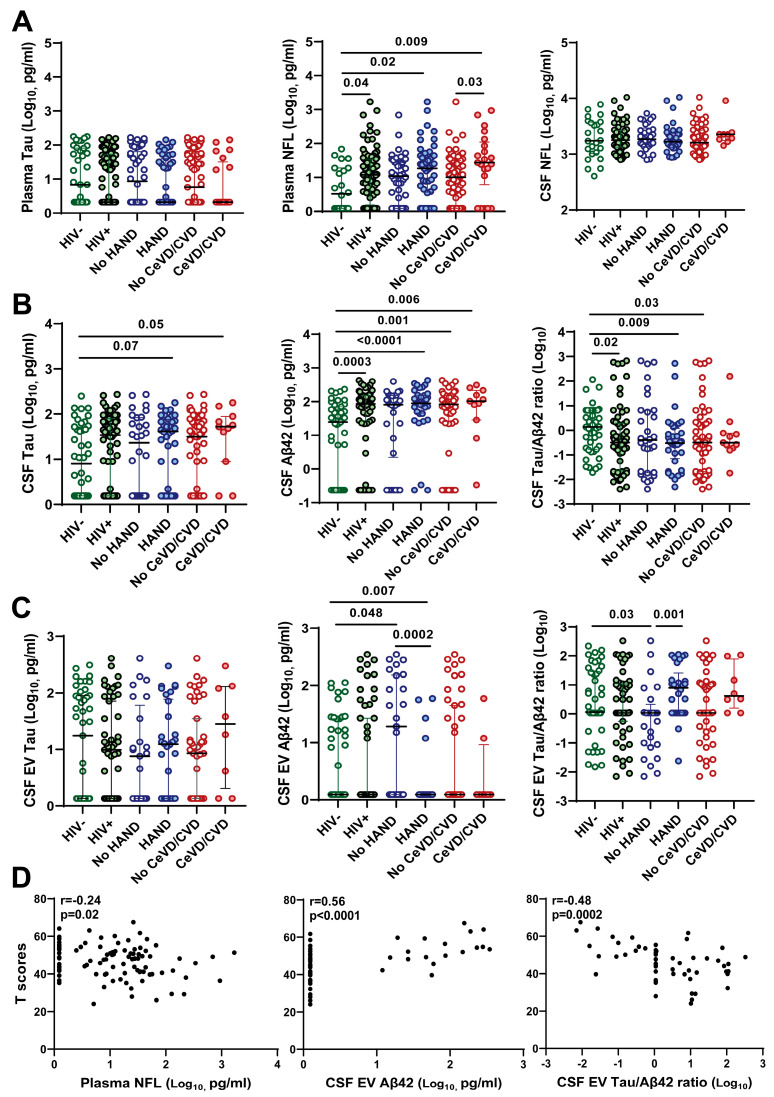
CSF extracellular vesicle Aβ42 and Tau/Aβ42 ratio are associated with HAND in PWH on ART. (**A**) Association of plasma Tau, plasma NFL, and CSF NFL with HIV infection, HAND, and vascular disease. (**B**) Association of CSF Tau, Aβ42, and Tau/Aβ42 ratio with HIV status, HAND, and vascular disease. (**C**) Association of CSF EV-associated Tau, Aβ42, and Tau/Aβ42 ratio with HIV status, HAND, and vascular disease. Medians and IQRs are indicated as horizontal and vertical lines, respectively. Statistical significance was calculated using the Mann–Whitney U test; significant differences (*p* < 0.05) are indicated. (**D**) Plasma NFL and CSF EV Tau/Aβ42 ratio correlate negatively, and CSF EV Aβ42 correlates positively with global neurocognitive T scores in PWH; Spearman’s rank correlation r and *p*-values are shown (significant correlations *p* < 0.05).

Next, we compared plasma and CSF soluble and EV-associated CNS injury and glial activation markers between groups by HIV, HAND, and vascular disease status (Figure 2). HIV infection was associated with increased plasma NFL (*p* = 0.04) and CSF Aβ42 (*p* = 0.0003) and a decreased CSF Tau/Aβ42 ratio (*p* = 0.02), but no significant difference was detected in plasma and CSF Tau (Figure 2A,B). Plasma and CSF NFL, CSF Aβ42, and CSF Tau/Aβ42 ratio had no significant associations with HAND. In contrast, CSF EV-associated Aβ42 levels were decreased (*p* = 0.0002) and CSF EV-associated Tau/Aβ42 ratio was increased (*p* = 0.001) in PWH with HAND vs. no HAND (Figure 2C). Plasma EV-associated Tau and Aβ42 were undetectable in most samples, and CSF EV-associated Tau had no significant associations with HIV status or HAND. There was a modest increase in plasma NFL (*p* = 0.03) and an increasing trend for the CSF EV-associated Tau/Aβ42 ratio (*p* = 0.1) in PWH with vs. without vascular disease. Given the differences in plasma NFL and CSF EV-associated Aβ42 and Tau/Aβ42 ratio by HAND status (Figure 2A,C), we performed correlation analyses of these biomarkers with global T scores. Plasma NFL and CSF EV-associated Tau/Aβ42 ratio correlated negatively, while CSF EV-associated Aβ42 correlated positively with global T scores (Figure 2D).

Increased GFAP has been associated with AD and other neurological diseases in previous studies [53,54]. Therefore, we also examined the associations of plasma, CSF, and CSF EV-associated GFAP with HIV status, HAND, and vascular disease (Supplemental Digital Content S2). Plasma, CSF, and CSF EV-associated GFAP levels were higher in PWH vs. HIV− controls (*p* = 0.007, <0.0001, and 0.0007, respectively). However, there was no significant difference by HAND or vascular disease status. Plasma GFAP had a weak negative correlation with global T scores (Spearman’s rank r = −0.22; *p* = 0.03) and correlated positively with plasma NFL (r = 0.41; *p* < 0.0001). As expected, plasma NFL and GFAP and CSF NFL, GFAP, and Tau correlated positively with age (Supplemental Digital Content S3).

### 3.4. Association of Decreased CSF EV Aβ42 and Increased CSF EV Tau/Aβ42 Ratio with Lower Neurocognitive Test Scores

Given the associations between CSF EV-associated Aβ42 and Tau/Aβ42 ratio, and plasma NFL or plasma GFAP and global T scores detected in the preceding analyses, we further examined these relationships in linear regression models adjusted for age (Table 2). These models were not adjusted for gender or race because global T scores were not significantly different between males vs. females (*p* = 0.57) or white vs. non-white (*p* = 0.46). Lower CSF EV-associated Aβ42 (β = 1.85, *p* < 0.0001) and higher CSF EV-associated Tau/Aβ42 ratio (β = −1.22, *p* = 0.0003) were associated with lower global neurocognitive T scores in these age-adjusted models. Plasma NFL showed a trend for negative association with global T scores (β = −0.65, *p* = 0.09), while plasma GFAP had no significant association when adjusted for age (*p* = 0.15). The optimal age-adjusted model included two biomarkers, CSF EV Aβ42 and plasma NFL, based on backward elimination to select the model with the lowest AIC values (236, 235, 418, and 232 for the full model, CSF EV Aβ42, plasma NFL, and both biomarkers, respectively).

To assess the relationship of CSF EV Aβ42 and CSF EV Tau/Aβ42 ratio to memory impairment (a hallmark of early aMCI/AD) versus cognitive domains more characteristic of HAND, 55 HIV+ individuals with available CSF EV Aβ42 and CSF EV Tau/Aβ42 ratio values were categorized in groups by CSF EV Aβ42 top/middle vs. bottom tertiles or CSF EV Tau/Aβ42 ratio bottom/middle vs. top tertiles. Groups according to these biomarker tertiles were similar in demographics, HIV−related characteristics, and frequency of the APOE4 genotype (Table 3). Compared with the top/middle tertiles, the CSF EV Aβ42 bottom tertile had a higher proportion of PWH with HAND (22% vs. 70%, respectively; *p* = 0.002) in both the ANI and MND categories (17% vs. 49% and 6% vs. 22%) and a trend for more frequent progression to HAD within 2.5 years (0% vs.16%). Next, we evaluated neurocognitive T scores in groups according to biomarker tertiles. Median T scores for all cognitive domains were significantly lower, and proportions with T scores < 40 were significantly higher, in PWH with HAND vs. no HAND; deficits in speed of information processing, attention/working memory, learning, memory, and motor domains were the most common findings (Supplemental Table S1). Median global and domain T scores were all significantly lower in the CSF EV Aβ42 bottom tertile group, with greatest differences in speed of information processing, attention/working memory, learning, and memory T scores and higher proportions with T scores <40 in speed of information processing (6% vs. 35%, *p* = 0.022), attention/working memory (0% vs. 35%, *p* = 0.005), memory (17% vs. 49%, *p* = 0.037), and motor (22% vs. 54%, *p* = 0.042) domains. Compared with the bottom/middle tertiles, the CSF EV Tau/Aβ42 ratio top tertile had a higher proportion of PWH with HAND (42% vs. 79%, respectively; *p* = 0.019) in both ANI and MND categories (31% vs. 53% and 11% vs. 21%) and a trend for more frequent progression to HAD within 2.5 years (6% vs. 21%). Median global and speed of information processing, attention/working memory, and motor domain T scores were significantly lower in the CSF EV Tau/Aβ42 ratio top tertile group, while abstraction/executive function, learning, memory, and verbal fluency T scores showed decreasing trends. The proportion with T scores <40 was significantly different only for attention/working memory domain (14% vs. 42%; *p* = 0.045), while speed of information processing, memory, and motor domains showed nonsignificant similar trends.

### 3.5. Association of Soluble but Not EV-Associated Vascular Injury Markers with HAND

In a recent study, we showed that soluble forms of vascular injury markers are increased in PWH with HAND compared with no HAND [55]. To determine if EV-associated forms of these markers have stronger associations with HAND compared to soluble forms, we examined the association of plasma EV-associated vascular injury markers (ICAM-1, VCAM-1, CRP) with HIV status, HAND, and vascular disease (Supplemental Digital Content S4). As expected, HIV infection and HAND were associated with increased plasma soluble ICAM-1 (*p* = 0.005 and 0.02, respectively), VCAM-1 (*p* = 0.02 and 0.004) and CRP (both *p* = 0.02). Plasma EV ICAM-1 and VCAM-1, but not CRP, were increased in PWH compared with HIV− controls, but there was no significant difference by HAND status. Thus, soluble forms of these vascular injury markers were more closely related to HAND than the corresponding EV-associated forms.

### 3.6. Comparative Abundance of Soluble vs. EV-Associated Biomarkers

To compare the relative abundance of soluble vs. EV-associated biomarkers in paired plasma and CSF samples, we used the Wilcoxon matched-pairs signed-rank test (Supplemental Digital Content S5). As expected, soluble Aβ42 and GFAP levels were higher in CSF vs. plasma, whereas ICAM-1, VCAM-1, and CRP levels were higher in plasma vs. CSF. Tau was detected in both plasma and CSF EVs, whereas Aβ42 and GFAP were detected only in CSF EVs. Aβ42 was more abundant in CSF vs. CSF EVs, while GFAP levels were not significantly different. Tau showed no consistent patterns when comparing relative levels of soluble vs. EV-associated forms. ICAM-1, VCAM-1, and CRP were more abundant in plasma vs. plasma EVs (*p* < 0.0001), whereas VCAM-1, CRP, and, to a lesser extent, ICAM-1 were more abundant in CSF EVs vs. CSF.

To further evaluate the association of Tau and Aβ42 with CSF EVs, we performed immunoblotting of CSF EVs following PK treatment to digest surface proteins while leaving internalized proteins intact (Supplemental Digital Content S6). For these experiments, we used pooled CSF from three PWH with HAND to isolate CSF EVs for immunoblotting and compared Tau and Aβ42 levels between lanes with vs. without PK treatment. Immunoblotting for CD9 and HSP70 was performed to detect the effects of PK treatment on exosome surface proteins (CD9) vs. internalized proteins (HSP70). Human frontal cortex gray matter tissue samples from two older PWH with HAND were included as positive controls for the detection of Tau and Aβ42 protein bands on blots. Tau was detected in CSF EVs in lanes with and without PK treatment, suggesting its localization inside EVs. In contrast, Aβ42 showed a loss of signal in the PK-treatment lane compared with the untreated control lane, suggesting that Aβ42—or aggregated forms of Aβ42—is associated with the outer surface of CSF EVs [36].

## 4. Discussion

Here, we report the first study of CSF EV Aβ42 and Tau levels in relation to cognitive impairment in virally suppressed PWH. In a cohort of PWH with a median age of 53 years, we show that decreased CSF EV Aβ42 levels and an increased CSF EV Tau/Aβ42 ratio, but not soluble forms of these markers, are associated with HAND and lower neurocognitive T scores. Consistent with previous studies [36,39,40], our findings suggest that Tau is localized inside CSF EVs, whereas Aβ42 or its aggregated forms may be associated with the surface of CSF EVs. Tau filaments may be tethered to the inner membrane of EVs, whereas Abeta42 localization on the surface of EVs may result from surface properties of soluble or oligomeric Abeta42 that mediate a high affinity for lipids on the surface of exosomes/EVs. The CSF EV Tau/Aβ42 ratio was more strongly associated with HAND than soluble or EV-associated Tau alone, consistent with studies that detected stronger associations between aMCI/AD and the CSF soluble Tau/Aβ42 ratio compared with the individual markers [22,23,24,56]. Aβ42 is an early marker whereas hyperphosphorylated Tau is a late marker of neurodegeneration in AD, and these markers interact synergistically in AD pathogenesis [56,57]. These findings suggest that CSF EV Aβ42 levels and Tau/Aβ42 ratio may help to distinguish aMCI/AD-related vs. HIV-related etiologies of cognitive decline in older PWH.

Given that the Tau/Aβ42 ratio is associated with an elevated risk of aMCI/AD in the general population [22], the decreased CSF EV Aβ42 levels and increased CSF EV Tau/Aβ42 ratio we observed in some PWH with HAND raises the possibility that these markers identify a subgroup with “HAND” that is due to non-HIV-related etiologies [30,50]. However, we observed a pattern of cognitive deficits more characteristic of HAND than aMCI (i.e., speed of information processing, attention/working memory, memory, and motor domains rather than specific deficits in learning/memory) [11,12,50] associated with these biomarkers, particularly decreased CSF EV Aβ42. Aging and chronic HIV infection can deregulate cellular processes, including mitochondrial activity and autophagy, which, in turn, may promote oxidative stress, protein misfolding, and abnormal accumulation of Aβ and Tau [18]. Hypoxia and vascular disease can also promote protein misfolding and aggregation. Large prospective studies of older PWH are needed to determine the prognostic significance of these biomarkers for predicting progression to aMCI/AD versus other etiologies of cognitive decline.

Although HAND was associated with decreased CSF EV Aβ42 levels and an increased CSF EV Tau/Aβ42 ratio in our study, we did not detect an association with soluble forms of these markers. Moreover, soluble Aβ42 and Tau/Aβ42 did not correlate with corresponding CSF EV-associated forms. Consistent with these findings, some studies reported no significant difference in Aβ deposition according to HIV serostatus or HAND stage [58,59], while others reported no significant difference in CSF Aβ peptides and p-Tau concentrations between groups stratified by HIV or HAND [26,60]. Unexpectedly, we observed higher CSF Aβ42 levels in PWH compared with HIV− controls; the explanation for this finding remains unclear and warrants further study. The association of total Tau levels with HIV or HAND remains controversial. Some studies reported an increased deposition of aggregated Tau in brain [61] or increased Tau in CSF [62,63,64], whereas others found no significant difference [19,27,65]. Further studies are needed to determine the specificity and diagnostic significance of Aβ and Tau biomarkers in PWH.

NFL is a sensitive biomarker of axonal injury in HAND and other neurological disorders [13,14]. In this study, mild forms of HAND were associated with plasma but not CSF NFL levels. The associations between plasma NFL and cognitive impairment were relatively weak, possibly reflecting milder forms of CNS injury/cognitive impairment in virally suppressed PWH on ART. CSF NFL is associated with HAD and correlates with viremia [13,15,66], but the association with HAND in virally suppressed PWH is less clear. In the present study, plasma NFL correlated negatively with global T scores, while plasma GFAP correlated positively with plasma NFL. Given that increased plasma GFAP may be an early marker of some amyloid-β and Tau-related pathologies [53,54], further studies are warranted to investigate its relationship to biotypes of cognitive impairment in PWH.

We found that soluble plasma vascular injury markers (ICAM-1, VCAM-1, and CRP) were associated with HAND, similar to findings in our recent study [55]. However, EV-associated forms of these markers did not show significant associations with HAND. These findings suggest that soluble vascular injury markers are more closely related to HAND than their corresponding EV-associated forms.

We acknowledge some limitations of the study. The sample size limited the statistical power to detect some associations, particularly for smaller subgroups. Additionally, the cohort was younger compared with most studies on aMCI/AD biomarkers and the majority of participants were white males, which limit the generalizability of our results. The limited volume of CSF available for EV isolation and the lower sensitivity of the Meso Scale Discovery assays compared with single-molecule technologies limited our ability to detect low levels of Tau and Aβ42 in EVs. Furthermore, we did not evaluate phosphorylated Tau, which is more closely related to neurodegeneration in AD than total Tau. Lastly, this was a cross-sectional study that included some PWH with pre-existing memory deficits. Given these limitations, some of the findings should be viewed as highly preliminary. Prospective studies are needed to evaluate CSF EV Aβ42 and Tau/Aβ42 ratio in larger cohorts representing diverse populations to determine the specificity and prognostic significance of these biomarkers for amyloid- and Tau-related pathologies.

## 5. Conclusions

In summary, we present evidence that mild forms of HAND are associated with decreased CSF EV Aβ42 levels and an increased CSF EV Tau/Aβ42 ratio and may relate to the risk of an aMCI/AD-related biotype of “HAND” that is not caused directly by HIV infection but is potentially impacted by HIV-associated factors such as chronic immune activation, neuroinflammation, oxidative stress, vascular disease, and ART exposure. Larger studies of older PWH representing diverse populations are needed to further evaluate the utility and prognostic significance of CSF EV-associated biomarkers in relation to aMCI/AD, HAND, and other neurological disorders.

## Figures and Tables

**Table 1:** Demographic and clinical characteristics of the study cohort.

	HIV− (*n* = 86)	HIV+ (*n* = 98)	No HAND (*n* = 47)	HAND * (*n* = 51)	*p*-Value
Age (years)	54 (46–61)	53 (47–59)	52 (47–57)	53 (49–61)	0.55
Male gender (*n*, %)	60 (70)	82 (84)	47 (100)	40 (78)	**0.0006**
Race (*n*, %) ^#^					0.68
Black	18 (28)	27 (28)	15 (32)	12 (24)	
White	46 (71)	68 (69)	31 (66)	37 (72)	
Other	1 (1)	3 (3)	1 (2)	2 (4)	
Current smoking (*n*, %)	27 (66)	48 (49)	19 (40)	29 (56.9)	0.11
Current alcohol use (*n*, %)		45 (46)	23 (49)	22 (43)	0.69
Diabetes (*n*, %)		16 (16)	6 (13)	10 (20)	0.42
Hypertension (*n*, %)		50 (51.0)	22 (47)	28 (55)	0.54
Hyperlipidemia (*n*, %)		28 (29)	10 (21)	18 (35)	0.18
Cerebrovascular disease/CVD (*n*, %)		23 (24)	5 (11)	18 (35)	**0.005**
Global neurocognitive T score		48 (41–53)	53 (50–57)	41 (37–44)	**<0.0001**
Global clinical rating		4 (3–5)	3 (2–4)	5 (5–6)	**<0.0001**
CSF protein		37 (31–47)	42 (33–52)	35 (27–44)	**0.04**
CSF WBC (cells/µL)		1 (1–3)	1 (0–3)	1 (1–3)	0.77
Duration of HIV infection (years)		15 (11–21)	15 (11–20)	15 (11–22)	0.89
Plasma viral load (<200 copies/mL)		94 (96)	45 (96)	49 (96)	1.00
CSF viral load (<50 copies/mL) ^Ϯ^		87 (96)	43 (98)	44 (94)	0.62
CD4 count (cells/µL)		540 (372–738)	589 (382–845)	537 (343–679)	0.41
Nadir CD4 count (cells/µL)		84 (14–223)	88 (12–262)	75 (16–183)	0.62
ART use (*n*, %)		98 (100)	47 (100)	51 (100)	1.00
Protease inhibitors		59 (60)	29 (62)	30 (59)	
Integrase inhibitors		19 (19)	9 (19)	10 (20)	
Duration of ART (years)		9 (5–14)	9 (5–14)	10 (5–14)	0.84

The median (interquartile range) is shown unless otherwise indicated. *p*-values for two-group comparisons between HAND and no HAND groups were calculated using chi-square or Fisher’s exact test for categorical variables and the Mann–Whitney U test for continuous variables. Bold font denotes *p* < 0.05. * HAND diagnoses were asymptomatic neurocognitive impairment (ANI, *n* = 27) or mild neurocognitive disorder (MND, *n* = 24). ^#^ Race and smoking data were not available for 21 and 45 HIV− controls, respectively. ^Ϯ^ CSF viral load data were not available for 7 HIV+ individuals. ART, antiretroviral therapy; CVD, cardiovascular disease; HAND, HIV-associated neurocognitive disorders; WBC, white blood cells.

**Table 2:** Association of CSF EV Aβ42, CSF EV Tau/Aβ42 ratio, plasma NFL, and plasma GFAP with global neurocognitive T scores in linear regression models adjusted for age.

Variable	β Coefficient	SE	95% CI	*p*-Value
*Single-marker models*				
CSF EV Aβ42	1.851	0.3873	1.074 to 2.628	<0.0001
CSF EV Tau/Aβ42 ratio	−1.218	0.3116	−1.843 to −0.593	0.0003
Plasma NFL	−0.6464	0.3862	−1.413 to 0.1206	0.098
Plasma GFAP	−0.9767	0.6751	−2.318 to 0.3641	0.15
*Optimal model*				
CSF EV Aβ42	1.824	0.372	1.077–2.571	<0.0001
Plasma NFL	−1.276	0.548	−2.377 to −0.175	0.024

Multivariable linear regression models were fit for global neurocognitive T scores in PWH on ART as the dependent variable, adjusting for age. Independent variables (CSF EV Aβ42 (*n* = 55), CSF EV Tau/Aβ42 ratio (*n* = 55), plasma NFL (*n* = 95), and plasma GFAP (*n* = 95)) were log2-transformed. The optimal model based on backward elimination and lowest AIC values included both CSF EV Aβ42 and plasma NFL (*n* = 55). SE, standard error; CI, confidence interval; EV, extracellular vesicle.

**Table 3:** Clinical characteristics and cognitive domain T scores by CSF EV Aβ42 and CSF EV Tau/Aβ42 ratio tertiles.

	CSF EV Ab42 Top/Middle Tertile (*n* = 18)	CSF EV Ab42 Bottom Tertile (*n* = 37)	*p*-Value	CSF EV Tau/Ab42 Ratio Bottom/Middle Tertile (*n* = 36)	CSF EV Tau/Ab42 Ratio Top Tertile (*n* = 19)	*p*-Value
Age (years)	53 [46, 57]	50 [47, 54]	0.83	52 [47, 57]	49 [44, 53]	0.43
Female gender (*n*, %)	1 (6)	8 (22)	0.26	3 (8)	6 (32)	0.067
Race (*n*, %)						0.68
Black	8 (44)	9 (24)	0.37	11 (31)	6 (32)	0.55
White	10 (56)	27 (73)		25 (69)	12 (63)	
Other	0 (0)	1 (3)		0 (0)	1 (6)	
Duration of HIV infection (years)	14 [9, 19]	14 [8, 22]	0.50	15 [9, 22]	14 [8, 17]	0.67
Nadir CD4 count (cells/µL)	181 [61, 256]	101 [21, 200]	0.17	146 [42, 223]	101 [39, 191]	0.68
CD4 count (cells/µL)	602 [463, 884]	537 [414, 706]	0.43	637 [448, 860]	506 [418, 603]	0.19
APOE4 carrier (*n*, %)	6 (35)	7 (28)	0.87	11 (39)	2 (14)	0.19
HAND diagnosis (*n*, %)	4 (22)	26 (70)	**0.0021**	15 (42)	15 (79)	**0.019**
HAND category (*n*, %)						
ANI	3 (17)	18 (49)	**0.0035**	11 (31)	10 (53)	**0.028**
MND	1 (6)	8 (22)		4 (11)	5 (26)	
Progression to HAD within 2.5 years (*n,* %)	0 (0)	6 (16)	0.20	2 (6)	4 (21)	0.21
Global T score	53 [49, 59]	44 [38, 50]	**<0.001**	50 [42, 55]	42 [35, 48]	**0.010**
Domain T score						
Abstraction/Executive function	55 [50, 61]	46 [40, 57]	**0.0090**	51 [46, 59]	45 [40, 54]	0.062
Speed of information processing	58 [51, 63]	45 [36, 50]	**<0.001**	51 [45, 60]	43 [31, 49]	**0.017**
Attention/Working memory	56 [52, 59]	44 [37, 50]	**<0.001**	52 [43, 57]	42 [36, 50]	**0.010**
Learning	53 [41, 58]	40 [34, 48]	**0.0021**	47 [37, 55]	40 [33, 45]	0.061
Memory	54 [45, 58]	42 [33, 48]	**0.0032**	48 [36, 55]	38 [32, 50]	0.056
Verbal fluency	54 [50, 60]	46 [41, 53]	**0.021**	52 [45, 59]	43 [39, 50]	0.053
Motor	50 [41, 57]	38 [31, 49]	**0.010**	48 [36, 55]	38 [26, 48]	**0.025**
Global T score < 40 (*n*, %)	1 (6)	14 (38)	**0.012**	8 (22)	7 (37)	0.401
Domain T score < 40 (*n*, %)						
Abstraction/Executive function	1 (6)	10 (27)	0.080	6 (17)	5 (26)	0.62
Speed of information processing	1 (6)	13 (35)	**0.022**	6 (17)	8 (42)	0.083
Attention/Working memory	0 (0)	13 (35)	**0.0049**	5 (14)	8 (42)	**0.045**
Learning	5 (28)	19 (51)	0.15	14 (39)	10 (53)	0.49
Memory	3 (17)	18 (49)	**0.037**	11 (31)	10 (53)	0.19
Verbal fluency	3 (17)	9 (24)	0.73	6 (17)	6 (32)	0.35
Motor	4 (22)	20 (54)	**0.042**	12 (33)	12 (63)	0.067

Medians [interquartile range] are shown unless otherwise indicated. *p*-values for binary comparisons between CSF EV Ab42 top/middle vs. bottom tertiles (>1.24 vs. ≤1.24 pg/mL (the limit of detection), respectively) or between CSF EV Tau/Ab42 ratio bottom/middle vs. top tertiles (≤8 and >8, respectively) were calculated using chi-square or Fisher’s exact test for categorical variables and the Mann–Whitney U test for continuous variables. Bold font denotes *p* < 0.05. *p*-values were not corrected for multiple comparisons. ANI, asymptomatic neurocognitive impairment; HAD, HIV-associated dementia; HAND, HIV-associated neurocognitive disorders; MND, mild neurocognitive disorder.

## Data Availability

All data generated or analyzed during this study are included in the published article and its Supplementary Information files or available from the corresponding author upon reasonable request.

## Supplementary Materials

The following supporting information can be downloaded at https://www.mdpi.com/article/10.3390/v16010072/s1, Supplemental Digital Content S1, Immunoblotting for exosome markers in plasma and CSF EV fractions; Supplemental Digital Content S2, Plasma but not CSF or CSF extracellular vesicle GFAP levels are associated with lower neurocognitive test scores and correlate with plasma NFL levels in PWH on ART; Supplemental Digital Content S3, Correlation of plasma and CSF Tau, NFL, and GFAP levels with age in PWH on ART; Supplemental Digital Content S4, Association of HAND with soluble but not extracellular vesicle-associated forms of ICAM-1, VCAM-1, and CRP; Supplemental Digital Content S5, Comparative abundance of biomarkers in plasma and CSF soluble and extracellular vesicle fractions; Supplemental Digital Content S6, Immunoblotting of Tau, Aβ42, HSP70, and CD9 in CSF extracellular vesicles; Table S1, Cognitive domain T scores by HAND status.

## References

[1] Aung H.L., Alagaratnam J., Chan P., Chow F.C., Joska J., Falutz J., Letendre S.L., Lin W., Munoz-Moreno J.A., Cinque P. (2023). Cognitive Health in Persons With Human Immunodeficiency Virus: The Impact of Early Treatment, Comorbidities, and Aging. J. Infect. Dis..

[2] Gabuzda D., Jamieson B.D., Collman R.G., Lederman M.M., Burdo T.H., Deeks S.G., Dittmer D.P., Fox H.S., Funderburg N.T., Pahwa S.G. (2020). Pathogenesis of Aging and Age-related Comorbidities in People with HIV: Highlights from the HIV ACTION Workshop. Pathog. Immun..

[3] Winston A., Spudich S. (2020). Cognitive disorders in people living with HIV. Lancet HIV.

[4] Saylor D., Dickens A.M., Sacktor N., Haughey N., Slusher B., Pletnikov M., Mankowski J.L., Brown A., Volsky D.J., McArthur J.C. (2016). HIV-associated neurocognitive disorder–pathogenesis and prospects for treatment. Nat. Rev. Neurol..

[5] Joseph S.B., Gianella S., Burdo T.H., Cinque P., Gisslen M., Letendre S., Nath A., Morgello S., Ndhlovu L.C., Spudich S. (2023). Biotypes of Central Nervous System Complications in People With Human Immunodeficiency Virus: Virology, Immunology, and Neuropathology. J. Infect. Dis..

[6] Aung H.L., Aghvinian M., Gouse H., Robbins R.N., Brew B.J., Mao L., Cysique L.A. (2021). Is There Any Evidence of Premature, Accentuated and Accelerated Aging Effects on Neurocognition in People Living with HIV? A Systematic Review. AIDS Behav..

[7] Jakabek D., Rae C.D., Brew B.J., Cysique L.A. (2022). Brain aging and cardiovascular factors in HIV: A longitudinal volume and shape MRI study. AIDS.

[8] Samboju V., Cobigo Y., Paul R., Naasan G., Hillis M., Tsuei T., Javandel S., Valcour V., Milanini B. (2021). Cerebrovascular Disease Correlates With Longitudinal Brain Atrophy in Virally Suppressed Older People Living With HIV. J. Acquir. Immune Defic. Syndr..

[9] Sanford R., Strain J., Dadar M., Maranzano J., Bonnet A., Mayo N.E., Scott S.C., Fellows L.K., Ances B.M., Collins D.L. (2019). HIV infection and cerebral small vessel disease are independently associated with brain atrophy and cognitive impairment. AIDS.

[10] Gabuzda D., Yankner B.A. (2013). Physiology: Inflammation links ageing to the brain. Nature.

[11] Rubin L.H., Sundermann E.E., Moore D.J. (2019). The current understanding of overlap between characteristics of HIV-associated neurocognitive disorders and Alzheimer’s disease. J. Neurovirology.

[12] Milanini B., Valcour V. (2017). Differentiating HIV-Associated Neurocognitive Disorders From Alzheimer’s Disease: An Emerging Issue in Geriatric NeuroHIV. Curr. HIV/AIDS Rep..

[13] Gisslen M., Price R.W., Andreasson U., Norgren N., Nilsson S., Hagberg L., Fuchs D., Spudich S., Blennow K., Zetterberg H. (2016). Plasma Concentration of the Neurofilament Light Protein (NFL) is a Biomarker of CNS Injury in HIV Infection: A Cross-Sectional Study. EBioMedicine.

[14] Yilmaz A., Blennow K., Hagberg L., Nilsson S., Price R.W., Schouten J., Spudich S., Underwood J., Zetterberg H., Gisslen M. (2017). Neurofilament light chain protein as a marker of neuronal injury: Review of its use in HIV-1 infection and reference values for HIV-negative controls. Expert. Rev. Mol. Diagn..

[15] McGuire J.L., Gill A.J., Douglas S.D., Kolson D.L., CNS HIV Anti-Retroviral Therapy Effects Research (CHARTER) group (2015). Central and peripheral markers of neurodegeneration and monocyte activation in HIV-associated neurocognitive disorders. J. Neurovirology.

[16] Guha D., Mukerji S.S., Chettimada S., Misra V., Lorenz D.R., Morgello S., Gabuzda D. (2019). Cerebrospinal fluid extracellular vesicles and neurofilament light protein as biomarkers of central nervous system injury in HIV-infected patients on antiretroviral therapy. AIDS.

[17] De Almeida S.M., Ribeiro C.E., Rotta I., Piovesan M., Tang B., Vaida F., Raboni S.M., Letendre S., Potter M., Batistela Fernandes M.S. (2018). Biomarkers of neuronal injury and amyloid metabolism in the cerebrospinal fluid of patients infected with HIV-1 subtypes B and C. J. NeuroVirology.

[18] Mackiewicz M.M., Overk C., Achim C.L., Masliah E. (2019). Pathogenesis of age-related HIV neurodegeneration. J. NeuroVirology.

[19] Gonzalez J., Wilson A., Byrd D., Cortes E.P., Crary J.F., Morgello S. (2023). Neuronal accumulation of hyperphosphorylated tau protein predicts stable memory impairment in people living with HIV. AIDS.

[20] Solomon I.H., De Girolami U., Chettimada S., Misra V., Singer E.J., Gabuzda D. (2017). Brain and liver pathology, amyloid deposition, and interferon responses among older HIV-positive patients in the late HAART era. BMC Infect. Dis..

[21] Hansson O., Seibyl J., Stomrud E., Zetterberg H., Trojanowski J.Q., Bittner T., Lifke V., Corradini V., Eichenlaub U., Batrla R. (2018). CSF biomarkers of Alzheimer’s disease concord with amyloid-β PET and predict clinical progression: A study of fully automated immunoassays in BioFINDER and ADNI cohorts. Alzheimers Dement..

[22] Li G., Sokal I., Quinn J.F., Leverenz J.B., Brodey M., Schellenberg G.D., Kaye J.A., Raskind M.A., Zhang J., Peskind E.R. (2007). CSF tau/Abeta42 ratio for increased risk of mild cognitive impairment: A follow-up study. Neurology.

[23] Park J.C., Han S.H., Yi D., Byun M.S., Lee J.H., Jang S., Ko K., Jeon S.Y., Lee Y.S., Kim Y.K. (2019). Plasma tau/amyloid-beta1-42 ratio predicts brain tau deposition and neurodegeneration in Alzheimer’s disease. Brain.

[24] Pereira J.B., Janelidze S., Stomrud E., Palmqvist S., van Westen D., Dage J.L., Mattsson-Carlgren N., Hansson O. (2021). Plasma markers predict changes in amyloid, tau, atrophy and cognition in non-demented subjects. Brain.

[25] Sundermann E.E., Campbell L.M., Villers O., Bondi M.W., Gouaux B., Salmon D.P., Galasko D., Soontornniyomkij V., Ellis R.J., Moore D.J. (2023). Alzheimer’s Disease Pathology in Middle Aged and Older People with HIV: Comparisons with Non-HIV Controls on a Healthy Aging and Alzheimer’s Disease Trajectory and Relationships with Cognitive Function. Viruses.

[26] Cooley S.A., Nelson B., Boerwinkle A., Yarasheski K.E., Kirmess K.M., Meyer M.R., Schindler S.E., Morris J.C., Fagan A., Ances B.M. (2023). Plasma Abeta42/Abeta40 Ratios in Older People With Human Immunodeficiency Virus. Clin. Infect. Dis..

[27] Cooley S.A., Strain J.F., Beaumont H., Boerwinkle A.H., Doyle J., Morris J.C., Benzinger T.L., Ances B.M. (2019). Tau Positron Emission Tomography Binding Is Not Elevated in HIV-Infected Individuals. J. Infect. Dis..

[28] Albrecht D.S., Sagare A., Pachicano M., Sweeney M.D., Toga A., Zlokovic B., Chui H., Joe E., Schneider L., Morris J.C. (2021). Early neuroinflammation is associated with lower amyloid and tau levels in cognitively normal older adults. Brain Behav. Immun..

[29] Fields J.A., Swinton M.K., Soontornniyomkij B., Carson A., Achim C.L. (2020). Beta amyloid levels in cerebrospinal fluid of HIV-infected people vary by exposure to antiretroviral therapy. AIDS.

[30] Lobo J.D., Moore D.J., Bondi M.W., Soontornniyomkij V., Soontornniyomkij B., Gouaux B., Achim C.L., Ellis R.J., Sundermann E.E. (2022). CSF markers of AD-related pathology relate specifically to memory impairment in older people with HIV: A pilot study. J. NeuroVirology.

[31] Morgello S., Cortes E.P., Gensler G., Meloni G., Jacobs M.M., Murray J., Borukov V., Crary J.F. (2021). HIV disease duration, but not active brain infection, predicts cortical amyloid beta deposition. AIDS.

[32] Mukerji S.S., Petersen K.J., Pohl K.M., Dastgheyb R.M., Fox H.S., Bilder R.M., Brouillette M.J., Gross A.L., Scott-Sheldon L.A.J., Paul R.H. (2023). Machine Learning Approaches to Understand Cognitive Phenotypes in People With HIV. J. Infect. Dis..

[33] Thompson A.G., Gray E., Heman-Ackah S.M., Mager I., Talbot K., Andaloussi S.E., Wood M.J., Turner M.R. (2016). Extracellular vesicles in neurodegenerative disease–pathogenesis to biomarkers. Nat. Rev. Neurol..

[34] Guha D., Lorenz D.R., Misra V., Chettimada S., Morgello S., Gabuzda D. (2019). Proteomic analysis of cerebrospinal fluid extracellular vesicles reveals synaptic injury, inflammation, and stress response markers in HIV patients with cognitive impairment. J. Neuroinflammation.

[35] Fiandaca M.S., Kapogiannis D., Mapstone M., Boxer A., Eitan E., Schwartz J.B., Abner E.L., Petersen R.C., Federoff H.J., Miller B.L. (2015). Identification of preclinical Alzheimer’s disease by a profile of pathogenic proteins in neurally derived blood exosomes: A case-control study. Alzheimers Dement..

[36] Lim C.Z.J., Zhang Y., Chen Y., Zhao H., Stephenson M.C., Ho N.R.Y., Chen Y., Chung J., Reilhac A., Loh T.P. (2019). Subtyping of circulating exosome-bound amyloid beta reflects brain plaque deposition. Nat. Commun..

[37] Pulliam L., Sun B., Mustapic M., Chawla S., Kapogiannis D. (2019). Plasma neuronal exosomes serve as biomarkers of cognitive impairment in HIV infection and Alzheimer’s disease. J. NeuroVirology.

[38] Coleman B.M., Hill A.F. (2015). Extracellular vesicles--Their role in the packaging and spread of misfolded proteins associated with neurodegenerative diseases. Semin. Cell Dev. Biol..

[39] Wang Y., Balaji V., Kaniyappan S., Kruger L., Irsen S., Tepper K., Chandupatla R., Maetzler W., Schneider A., Mandelkow E. (2017). The release and trans-synaptic transmission of Tau via exosomes. Mol. Neurodegener..

[40] Ruan Z., Pathak D., Venkatesan Kalavai S., Yoshii-Kitahara A., Muraoka S., Bhatt N., Takamatsu-Yukawa K., Hu J., Wang Y., Hersh S. (2021). Alzheimer’s disease brain-derived extracellular vesicles spread tau pathology in interneurons. Brain.

[41] Winston C.N., Goetzl E.J., Akers J.C., Carter B.S., Rockenstein E.M., Galasko D., Masliah E., Rissman R.A. (2016). Prediction of conversion from mild cognitive impairment to dementia with neuronally derived blood exosome protein profile. Alzheimers Dement..

[42] Jia L., Qiu Q., Zhang H., Chu L., Du Y., Zhang J., Zhou C., Liang F., Shi S., Wang S. (2019). Concordance between the assessment of Abeta42, T-tau, and P-T181-tau in peripheral blood neuronal-derived exosomes and cerebrospinal fluid. Alzheimers Dement..

[43] Chettimada S., Lorenz D.R., Misra V., Dillon S.T., Reeves R.K., Manickam C., Morgello S., Kirk G.D., Mehta S.H., Gabuzda D. (2018). Exosome markers associated with immune activation and oxidative stress in HIV patients on antiretroviral therapy. Sci. Rep..

[44] Kodidela S., Gerth K., Haque S., Gong Y., Ismael S., Singh A., Tauheed I., Kumar S. (2019). Extracellular Vesicles: A Possible Link between HIV and Alzheimer’s Disease-Like Pathology in HIV Subjects?. Cells.

[45] Kodidela S., Gerth K., Sinha N., Kumar A., Kumar P., Kumar S. (2020). Circulatory Astrocyte and Neuronal EVs as Potential Biomarkers of Neurological Dysfunction in HIV-Infected Subjects and Alcohol/Tobacco Users. Diagnostics.

[46] Hu G., Yang L., Cai Y., Niu F., Mezzacappa F., Callen S., Fox H.S., Buch S. (2016). Emerging roles of extracellular vesicles in neurodegenerative disorders: Focus on HIV-associated neurological complications. Cell Death Dis..

[47] Heaton R.K., Clifford D.B., Franklin D.R., Woods S.P., Ake C., Vaida F., Ellis R.J., Letendre S.L., Marcotte T.D., Atkinson J.H. (2010). HIV-associated neurocognitive disorders persist in the era of potent antiretroviral therapy: CHARTER Study. Neurology.

[48] Morgello S., Gelman B.B., Kozlowski P.B., Vinters H.V., Masliah E., Cornford M., Cavert W., Marra C., Grant I., Singer E.J. (2001). The National NeuroAIDS Tissue Consortium: A new paradigm in brain banking with an emphasis on infectious disease. Neuropathol. Appl. Neurobiol..

[49] Woods S.P., Rippeth J.D., Frol A.B., Levy J.K., Ryan E., Soukup V.M., Hinkin C.H., Lazzaretto D., Cherner M., Marcotte T.D. (2004). Interrater reliability of clinical ratings and neurocognitive diagnoses in HIV. J. Clin. Exp. Neuropsychol..

[50] Sundermann E.E., Bondi M.W., Campbell L.M., Gouaux B., Moore R.C., Soontornniyomkij V., Moore D.J. (2021). Distinguishing Amnestic Mild Cognitive Impairment From HIV-Associated Neurocognitive Disorders. J. Infect. Dis..

[51] Antinori A., Arendt G., Becker J.T., Brew B.J., Byrd D.A., Cherner M., Clifford D.B., Cinque P., Epstein L.G., Goodkin K. (2007). Updated research nosology for HIV-associated neurocognitive disorders. Neurology.

[52] Lorenz D.R., Mukerji S.S., Misra V., Uno H., Gelman B.B., Moore D.J., Singer E.J., Morgello S., Gabuzda D. (2021). Multimorbidity networks associated with frailty among middle-aged and older people with HIV. AIDS.

[53] Chatterjee P., Pedrini S., Stoops E., Goozee K., Villemagne V.L., Asih P.R., Verberk I.M.W., Dave P., Taddei K., Sohrabi H.R. (2021). Plasma glial fibrillary acidic protein is elevated in cognitively normal older adults at risk of Alzheimer’s disease. Transl. Psychiatry.

[54] Pereira J.B., Janelidze S., Smith R., Mattsson-Carlgren N., Palmqvist S., Teunissen C.E., Zetterberg H., Stomrud E., Ashton N.J., Blennow K. (2021). Plasma GFAP is an early marker of amyloid-beta but not tau pathology in Alzheimer’s disease. Brain.

[55] Guha D., Misra V., Yin J., Horiguchi M., Uno H., Gabuzda D. (2023). Vascular injury markers associated with cognitive impairment in people with HIV on suppressive antiretroviral therapy. AIDS.

[56] Busche M.A., Hyman B.T. (2020). Synergy between amyloid-beta and tau in Alzheimer’s disease. Nat. Neurosci..

[57] Pereira J.B., Janelidze S., Ossenkoppele R., Kvartsberg H., Brinkmalm A., Mattsson-Carlgren N., Stomrud E., Smith R., Zetterberg H., Blennow K. (2021). Untangling the association of amyloid-beta and tau with synaptic and axonal loss in Alzheimer’s disease. Brain.

[58] Mohamed M., Skolasky R.L., Zhou Y., Ye W., Brasic J.R., Brown A., Pardo C.A., Barker P.B., Wong D.F., Sacktor N. (2020). Beta-amyloid (Abeta) uptake by PET imaging in older HIV+ and HIV- individuals. J. Neurovirology.

[59] Howdle G.C., Quide Y., Kassem M.S., Johnson K., Rae C.D., Brew B.J., Cysique L.A. (2020). Brain amyloid in virally suppressed HIV-associated neurocognitive disorder. Neurol. Neuroimmunol. Neuroinflammation.

[60] Peterson J., Gisslen M., Zetterberg H., Fuchs D., Shacklett B.L., Hagberg L., Yiannoutsos C.T., Spudich S.S., Price R.W. (2014). Cerebrospinal fluid (CSF) neuronal biomarkers across the spectrum of HIV infection: Hierarchy of injury and detection. PLoS ONE.

[61] Anthony I.C., Ramage S.N., Carnie F.W., Simmonds P., Bell J.E. (2006). Accelerated Tau deposition in the brains of individuals infected with human immunodeficiency virus-1 before and after the advent of highly active anti-retroviral therapy. Acta Neuropathol..

[62] Brew B.J., Pemberton L., Blennow K., Wallin A., Hagberg L. (2005). CSF amyloid beta42 and tau levels correlate with AIDS dementia complex. Neurology.

[63] Gisslen M., Krut J., Andreasson U., Blennow K., Cinque P., Brew B.J., Spudich S., Hagberg L., Rosengren L., Price R.W. (2009). Amyloid and tau cerebrospinal fluid biomarkers in HIV infection. BMC Neurol..

[64] Ellis R.J., Chenna A., Petropoulos C.J., Lie Y., Curanovic D., Crescini M., Winslow J., Sundermann E., Tang B., Letendre S.L. (2022). Higher cerebrospinal fluid biomarkers of neuronal injury in HIV-associated neurocognitive impairment. J. Neurovirology.

[65] Clifford D.B., Fagan A.M., Holtzman D.M., Morris J.C., Teshome M., Shah A.R., Kauwe J.S. (2009). CSF biomarkers of Alzheimer disease in HIV-associated neurologic disease. Neurology.

[66] Gisslen M., Keating S.M., Spudich S., Arechiga V., Stephenson S., Zetterberg H., Di Germanio C., Blennow K., Fuchs D., Hagberg L. (2021). Compartmentalization of cerebrospinal fluid inflammation across the spectrum of untreated HIV-1 infection, central nervous system injury and viral suppression. PLoS ONE.

