# Supplementary material for: CSF Extracellular Vesicle Aβ42 and Tau/Aβ42 Ratio Are Associated with Cognitive Impairment in Older People with HIV
Source: Viruses. 2023 Dec 31;16(1):72. doi: 10.3390/v16010072 (PMC10818296; doi:10.3390/v16010072)
Supplement: Supplementary file 1 [file viruses-16-00072-s001.zip › Supplemental Digital Content S1-S6.pdf]

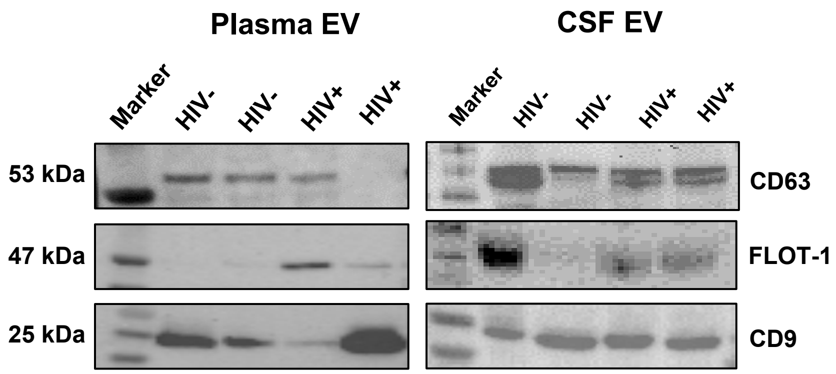

Supplemental Digital Content 1. Immunoblotting for exosome markers in plasma and CSF EV fractions. Shown are exosome markers CD63, CD9, and Flotillin-1 (FLOT-1) detected by immunoblotting of plasma and CSF EVs from two representative HIV- and two representative HIV+ individuals.

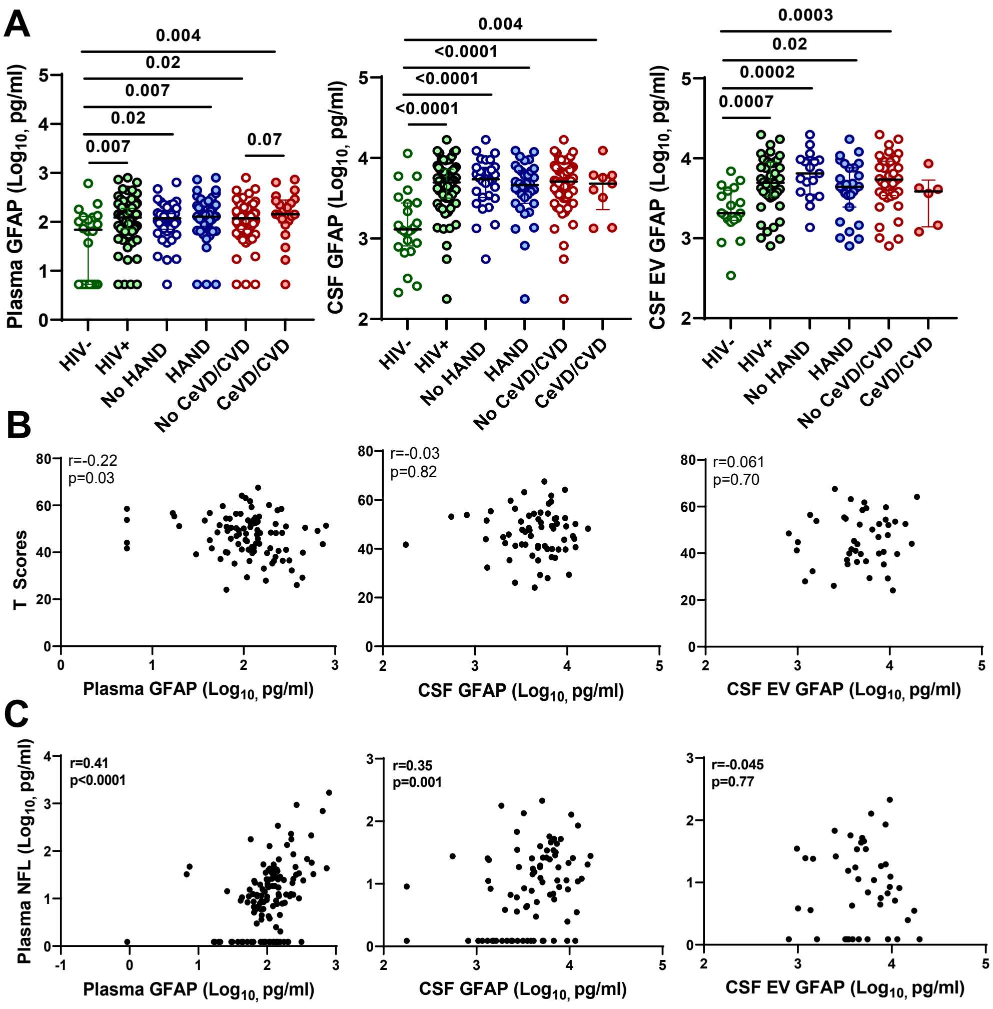

Supplemental Digital Content 2. Plasma but not CSF or CSF extracellular vesicle GFAP levels are associated with lower neurocognitive test scores and correlate with plasma NFL levels in PWH on ART. (A) Association of plasma, CSF, and CSF EV GFAP levels with HIV status, HAND, and vascular disease. (B) Plasma but not CSF or CSF EV GFAP levels correlate negatively with global neurocognitive T scores. (C) GFAP levels in plasma and CSF but not CSF EVs correlate positively with plasma NFL. Relationships between continuous variables were analyzed by Spearman's rank correlation (significant correlations $p<0.05$)

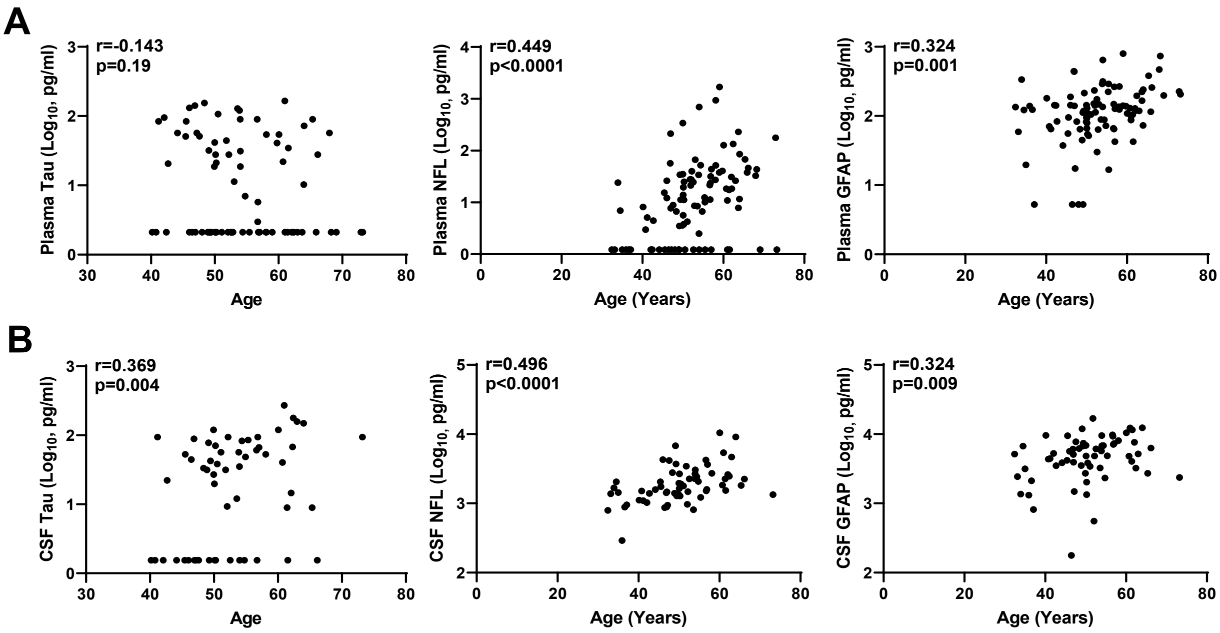

Supplemental Digital Content 3. Correlation of plasma and CSF Tau, NFL, and GFAP levels with age in PWH on ART. Plasma NFL and GFAP (A) and CSF Tau, NFL, and GFAP levels (B) correlate positively with age. Relationships between continuous variables were analyzed by Spearman's rank correlation (significant correlations $p<0.05$).

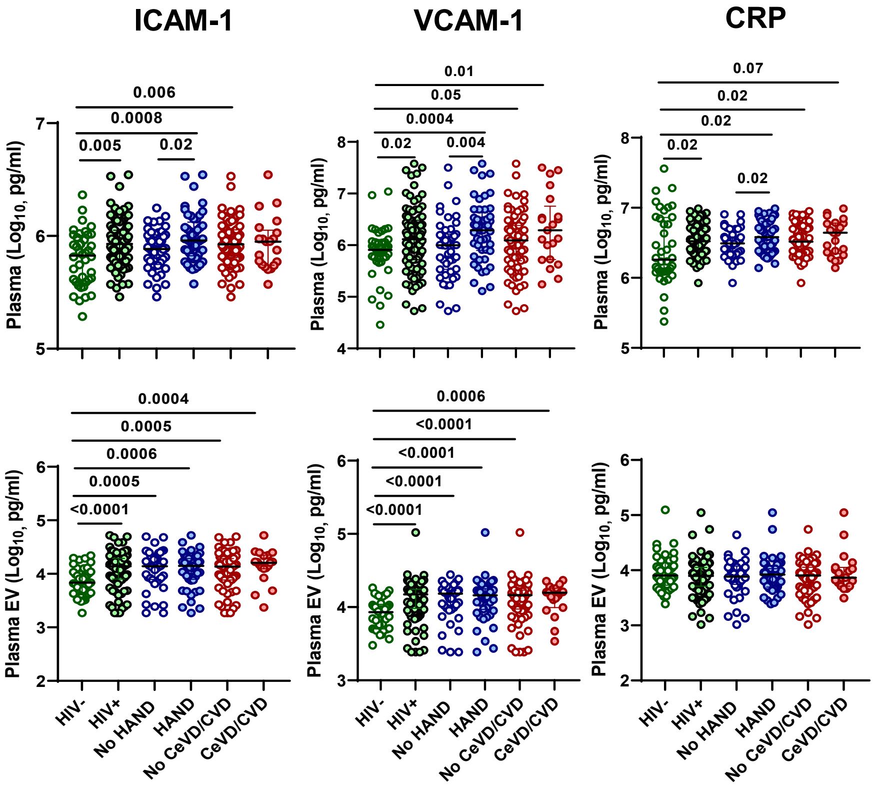

Supplemental Digital Content 4. Association of HAND with soluble but not extracellular vesicle-associated forms of ICAM-1, VCAM-1, and CRP. Association of plasma (top panels) or plasma EV (bottom panels) ICAM-1, VCAM-1, and CRP levels with HIV status, HAND, and vascular disease. Association of plasma EV ICAM-1, VCAM-1, and CRP with HIV infection, HAND, and vascular disease (bottom panels). Medians and IQRs are indicated as horizontal and vertical lines, respectively. Statistical significance calculated using Mann–Whitney U test; significant differences ($p<0.05$) are indicated.

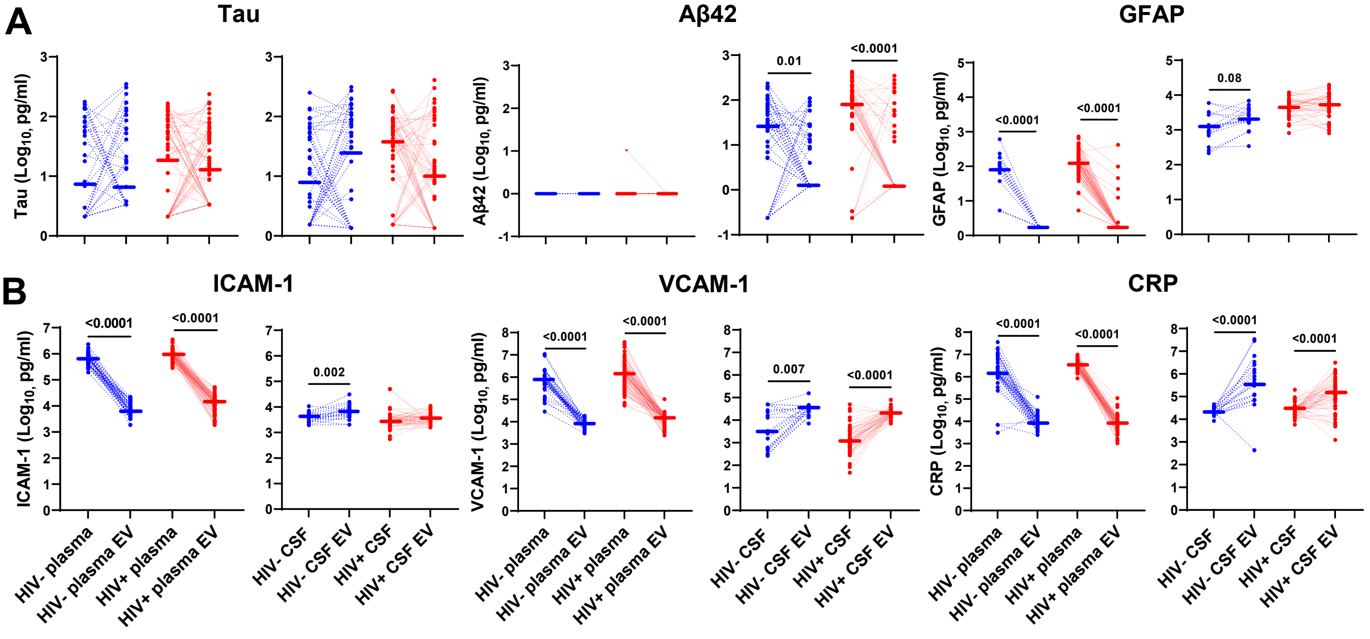

Supplemental Digital Content 5. Comparative abundance of biomarkers in plasma and CSF soluble and extracellular vesicle fractions. (A) Comparison of plasma and CSF soluble Tau, Aβ42, and GFAP levels with those detected in equivalent volumes of corresponding plasma and CSF EV fractions in HIV- and HIV+ individuals. (B) Comparison of plasma and CSF vascular injury marker levels (ICAM-1, VCAM-1, CRP) with those in corresponding EV fractions. Medians are indicated as horizontal lines. Statistical significance was calculated using Wilcoxon matched pairs signed rank test; significant differences ($p<0.05$) are indicated.

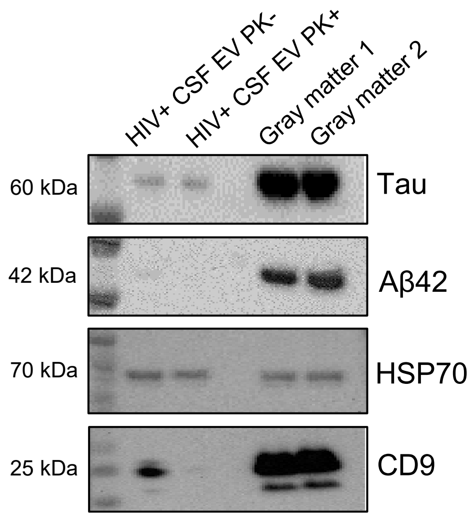

Supplemental Digital Content 6. Immunoblotting of Tau, Aβ42, HSP70, and CD9 in CSF extracellular vesicles. CSF EVs were isolated from pooled HIV+ CSF (n=3 HIV+ individuals with HAND, 150 µl CSF from each donor) and pooled CSF EV sample split into equal parts and then incubated with or without proteinase K (PK+ and PK-, respectively) as described in the Methods prior to immunoblotting. As positive controls for detection of Tau and Aβ42 protein bands on immunoblots, we included samples of frozen frontal cortex gray matter tissue samples (~ 30 mg) obtained at autopsy from 2 HIV+ individuals over age 60 and lysed in RIPA buffer with protease inhibitors (lanes 3 and 4). As additional controls, we performed immunoblotting for exosome markers CD9 and HSP70 to detect the effects of proteinase K treatment on exosome surface proteins (CD9) vs. internalized proteins (HSP70). Results are representative of two to three independent experiments.
