# Supplementary material for: CSF Extracellular Vesicle Aβ42 and Tau/Aβ42 Ratio Are Associated with Cognitive Impairment in Older People with HIV
Source: Viruses. 2023 Dec 31;16(1):72. doi: 10.3390/v16010072 (PMC10818296; doi:10.3390/v16010072)
Supplement: Supplementary file 1 [file viruses-16-00072-s001.zip › Supplemental Table S1.pdf]

Supplemental Table 1. Cognitive domain T scores by HAND status

	All HIV+ (n=98)	No HAND (n=47)	HAND (n=51)	P-value
Domain T score				
Abstraction/Executive function	51 [44, 59]	58 [52, 62]	45 [41, 51]	**<0.001**
Speed of information processing	50 [42, 57]	57 [51, 60]	42 [36, 50]	**<0.001**
Attention/Working memory	49 [41, 54]	52 [50, 58]	42 [38, 48]	**<0.001**
Learning	45 [37, 52]	52 [46, 59]	39 [32, 43]	**<0.001**
Memory	45 [36, 53]	51 [46, 56]	37 [32, 44]	**<0.001**
Verbal fluency	51 [43, 58]	57 [51, 60]	43 [39, 51]	**<0.001**
Motor	45 [36, 52]	50 [44, 57]	36 [31, 47]	**<0.001**
Domain T score < 40 (n, %)				
Abstraction/Executive function	14 (14)	2 (4)	12 (24)	**0.017**
Speed of information processing	21 (22)	0 (0)	21 (41)	**<0.001**
Attention/Working memory	20 (20)	1 (2)	19 (37)	**<0.001**
Learning	35 (36)	4 (9)	31 (61)	**<0.001**
Memory	35 (36)	4 (9)	31 (61)	**<0.001**
Verbal fluency	19 (19)	2 (4)	17 (33)	**<0.001**
Motor	38 (39)	5 (11)	33 (65)	**<0.001**

Medians (interquartile range) are shown unless otherwise indicated. P-values for binary comparisons between HAND vs. no HAND groups were calculated using chi-square or Fisher's exact test for categorical variables and Mann-Whitney U test for continuous variables. Bold font denotes p <0.05. HAND, HIV-associated neurocognitive disorders.
